# Autocrine Signaling Underlies Fast Repetitive Plasma Membrane Translocation of Conventional and Novel Protein Kinase C Isoforms in β Cells*

**DOI:** 10.1074/jbc.M115.698456

**Published:** 2016-05-20

**Authors:** Anne Wuttke, Qian Yu, Anders Tengholm

**Affiliations:** From the Department of Medical Cell Biology, Uppsala University, Biomedical Centre, Box 571, 75123 Uppsala, Sweden

**Keywords:** β cell, calcium, diacylglycerol, exocytosis, PKC, purinergic receptor, oscillations

## Abstract

PKC signaling has been implicated in the regulation of many cell functions, including metabolism, cell death, proliferation, and secretion. Activation of conventional and novel PKC isoforms is associated with their Ca^2+^- and/or diacylglycerol (DAG)-dependent translocation to the plasma membrane. In β cells, exocytosis of insulin granules evokes brief (<10 s) local DAG elevations (“spiking”) at the plasma membrane because of autocrine activation of P2Y_1_ purinoceptors by ATP co-released with insulin. Using total internal reflection microscopy, fluorescent protein-tagged PKCs, and signaling biosensors, we investigated whether DAG spiking causes membrane recruitment of PKCs and whether different classes of PKCs show characteristic responses. Glucose stimulation of MIN6 cells triggered DAG spiking with concomitant repetitive translocation of the novel isoforms PKCδ, PKCϵ, and PKCη. The conventional PKCα, PKCβI, and PKCβII isoforms showed a more complex pattern with both rapid and slow translocation. K^+^ depolarization-induced PKCϵ translocation entirely mirrored DAG spiking, whereas PKCβI translocation showed a sustained component, reflecting the subplasma membrane Ca^2+^ concentration ([Ca^2+^]_pm_), with additional effect during DAG spikes. Interference with DAG spiking by purinoceptor inhibition prevented intermittent translocation of PKCs and reduced insulin secretion but did not affect [Ca^2+^]_pm_ elevation or sustained PKCβI translocation. The muscarinic agonist carbachol induced pronounced transient PKCβI translocation and sustained recruitment of PKCϵ. When rise of [Ca^2+^]_pm_ was prevented, the carbachol-induced DAG and PKCϵ responses were somewhat reduced, but PKCβI translocation was completely abolished. We conclude that exocytosis-induced DAG spikes efficiently recruit both conventional and novel PKCs to the β cell plasma membrane. PKC signaling is thus implicated in autocrine regulation of β cell function.

## Introduction

PKC is a serine/threonine kinase important for a broad range of cellular processes (1, 2). The PKC family contains 10 isoforms that are divided into three groups depending on their mechanism of activation. Conventional PKCs (cPKCs)^2^ (PKCα, βI, βII, and γ) are activated by diacylglycerol (DAG) and Ca^2+^. Novel PKCs (nPKCs) (PKCδ, ϵ, η, and θ) respond to DAG but not to Ca^2+^. The atypical isoforms (aPKCs) (PKCζ and ι/λ) are independent of both DAG and Ca^2+^ (1, 2). Pancreatic β cells, which play a pivotal role in glucose homeostasis by releasing insulin, express members of all three PKC families. There is evidence that PKCα, βII, δ, ϵ, ζ, and ι/λ are expressed whereas the γ isoform is not (3–10). Conflicting results have been reported regarding expression of the βI, η, and θ isoforms (3–6, 8–10), perhaps reflecting differences in species, cell lines, and methodology to examine expression.

PKCs are involved in various aspects of β cell function, like proliferation, differentiation, and death, as well as insulin secretion (11), but the precise role of various isoforms is difficult to define because of a lack of selective pharmacological tools and potential problems with compensatory mechanisms and functional redundancy in genetic ablation studies. PKC-activating phorbol esters were found early to stimulate insulin secretion (12, 13), an effect mediated by sensitization of the secretory machinery to Ca^2+^ (14, 15). Although the involvement of PKC in the regulation of insulin release by G protein-coupled receptor stimuli is well established (11), its role in glucose-stimulated secretion is controversial. Down-regulation of PKC activity has little effect on the secretory response to glucose (16, 17), and experiments with PKC inhibitors have yielded conflicting results (18–23). Studies with genetic ablation of different PKC isozymes indicate that PKCδ and ι/λ are important for insulin secretion either by direct effects on the exocytosis machinery (24) or by controlling the expression of genes important for β cell differentiation (4). However, adenoviral overexpression of wild-type and kinase-dead forms of PKCα and δ failed to affect glucose-stimulated insulin secretion in another study (18). Also, the functional importance of PKCϵ is unclear. Expression of a dominant negative mutant suppressed exocytosis in isolated β cells (25), and a specific PKC translocation inhibitor reduced insulin secretion from rat islets (21). However, functional ablation of the protein did not affect glucose-induced insulin secretion and even amplified that from islets treated with fatty acids (26).

Activation of conventional and nPKCs typically involves their translocation to the plasma membrane. For nPKCs, this process is mostly DAG-driven, and for cPKCs, it depends on a combination of Ca^2+^ and DAG dynamics (27, 28). We recently discovered that glucose induces rapid DAG elevations in restricted regions of the β cell plasma membrane with durations of <10 s. They reflect the exocytotic release of adenine nucleotides with autocrine feedback activation of P2Y_1_ purinoceptors (29). Moreover, these DAG microdomains induced PKC activation, measured as translocation of fluorescence-labeled myristoylated alanine-rich C-kinase substrate (MARCKS). However, it remains unclear whether the DAG microdomains trigger PKC translocation or whether DAG spiking merely activates PKCs already present at the plasma membrane. It also remains to be establish which subclasses of PKCs are activated and whether the different isoforms respond differentially to the glucose-induced DAG signaling pattern. Therefore, we analyzed the translocation dynamics of various fluorescence-tagged PKC isoforms and DAG dynamics as well as the subplasma membrane Ca^2+^ concentration ([Ca^2+^]_pm_) in insulin-secreting β cells using total internal reflection fluorescence (TIRF) microscopy.

## Results

### 

#### 

##### Depolarization-induced DAG Spiking Triggers nPKC-dependent MARCKS Phosphorylation

**FIGURE 1.**
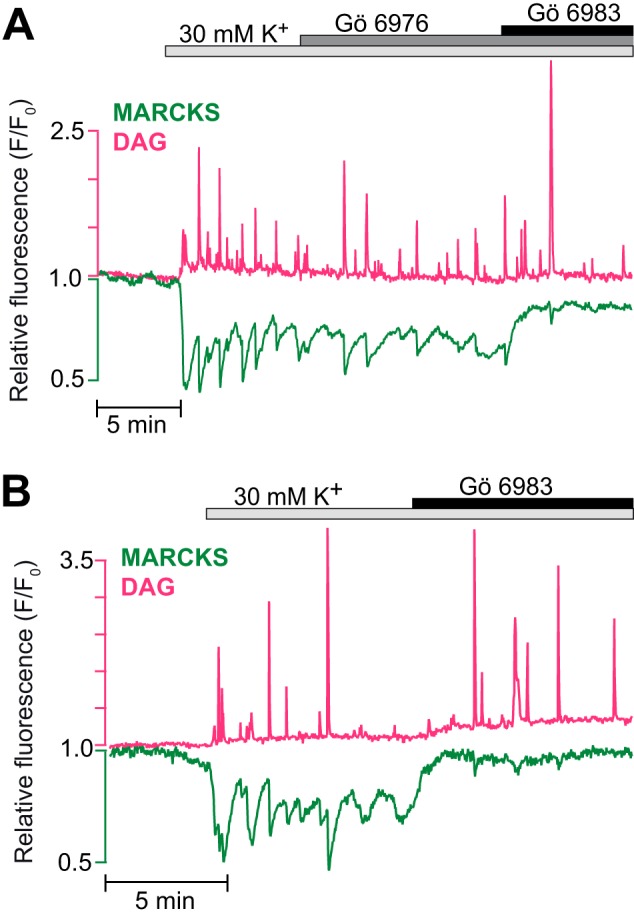
**Fast, repetitive MARCKS phosphorylation is mediated by novel PKCs.** *A* and *B*, simultaneous TIRF microscopy recordings of plasma membrane DAG concentration (*magenta*) and membrane localization of MARCKS-GFP (*green*) in single MIN6 cells. Membrane depolarization with 30 mm K^+^ induced DAG spiking, which was paralleled by transient drops of MARCKS fluorescence, representing short-lived PKC activity that resulted in MARCKS phosphorylation and dissociation from the membrane. MARCKS transients were not affected by 1 μm cPKC inhibitor Gö 6976 (*A*) but vanished in response to 1 μm Gö 6983, an inhibitor of both conventional and nPKCs, when added in the presence (*A*) or absence (*B*) of Gö 6976. *n* = 14 cells in three experiments for Gö 6976 and 15 cells in five experiments for Gö 6983.

TIRF imaging of MIN6 insulinoma cells co-expressing an mCherry-tagged DAG sensor and the PKC activity detector MARCKS-GFP showed that K^+^-mediated membrane depolarization induced repetitive, brief (<10 s) and pronounced DAG elevations. Each DAG spike was associated with less rapid dissociation of MARCKS-GFP from the plasma membrane (Fig. 1, *A* and *B*). Gö 6976, an inhibitor of cPKCs, had little effect on these responses, whereas Gö 6983, targeting both conventional and nPKCs, immediately suppressed MARCKS-GFP translocation without affecting DAG spiking (Fig. 1, *A* and *B*). These data indicate that DAG spiking primarily activates nPKCs in β cells.

##### Glucose-induced Plasma Membrane Translocation of nPKCs Reflects DAG Spiking

**FIGURE 2.**
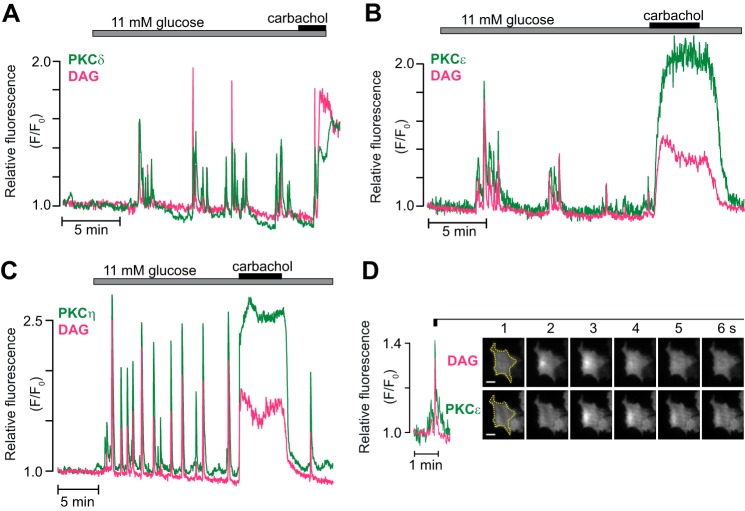
**Glucose- and carbachol-induced translocation of nPKCs.** Representative TIRF microscopy recordings from single MIN6-cells co-expressing the DAG biosensor (*magenta*) and GFP-labeled PKC (*green*) during stimulation with an increase in glucose concentration from 3 to 11 mm, followed by addition of 100 μm carbachol. *A–C*, DAG dynamics and translocation of PKCδ (*A*, *n* = 7 cells in three experiments), PKCϵ (*B*, *n* = 8 cells in four experiments), and PKCη (*C*, *n* = 9 cells in three experiments). *D*, TIRF image pairs acquired every second showing the spatial distribution of a DAG spike and corresponding PKCϵ translocation. The cell border is outlined in *yellow*. The DAG spike occurs in a restricted part of the plasma membrane, and PKCϵ translocates to the same region. *Scale bars* = 5 μm.

**FIGURE 3.**
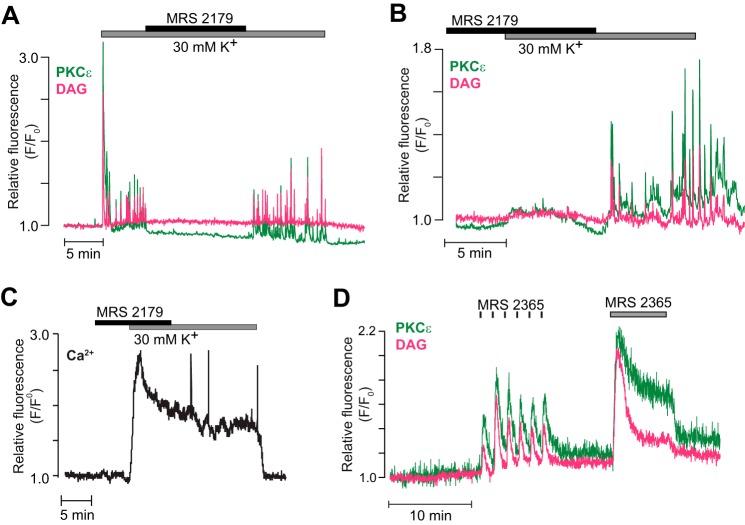
**The depolarization-induced PKCϵ translocation pattern reflects DAG dynamics.** Representative TIRF microscopy recordings from single MIN6 cells co-expressing the DAG biosensor (*magenta*) and GFP-labeled PKCϵ (*green*) or expressing the Ca^2+^ sensor R-GECO (*black*). *A* and *B*, DAG dynamics and PKCϵ translocation in cells exposed to 3 mm glucose, 30 mm K^+^, and 10 μm of the P2Y_1_ receptor antagonist MRS 2179. *n* = 6 cells in two experiments (*A*) and 7 cells in three experiments (*B*). *C*, single-cell recording of the [Ca^2+^]_pm_ response to depolarization with high K^+^ in the presence of MRS 2179 (*n* = 15 cells from three experiments). *D*, simultaneous recording of DAG dynamics and PKCϵ translocation in response to repetitive applications of the P2Y_1_ receptor agonist MRS 2365 (*n* = 14 cells from five experiments).

MIN6-cells were next co-transfected with the DAG biosensor and different GFP-tagged PKC isoforms. All nPKCs tested (δ, ϵ, and η) showed rapid, transient, and repetitive glucose-induced translocation between the cytoplasm and the plasma membrane in response to glucose, whereas the muscarinic agonist carbachol induced sustained membrane association, almost perfectly mirroring simultaneously measured DAG patterns (Fig. 2, *A–C*). The glucose-induced DAG spikes were often spatially confined, and the nPKCs translocated specifically to the membrane regions with elevated DAG, as illustrated for PKCϵ in Fig. 2*D*. PKCϵ was further investigated to evaluate the DAG dependence of the PKC translocation. Like glucose stimulation, membrane depolarization with a high K^+^ concentration resulted in parallel DAG spiking and PKCϵ-GFP translocation (Fig. 3*A*). Secretagogue-induced DAG spiking in β cells is due to exocytotic release of ATP with autocrine feedback activation of P2Y_1_ purinoceptors, which in turn activates phospholipase C (29). Consistent with DAG spiking underlying the brief plasma membrane binding of PKCϵ, both events were prevented by the P2Y_1_ receptor inhibitor MRS 2179 (Fig. 3*A*). Similarly, introduction of MRS 2179 before K^+^ depolarization prevented the appearance of DAG spikes as well as the concomitant transient PKCϵ translocation (Fig. 3*B*) without affecting the sustained depolarization-induced increase of [Ca^2+^]_pm_ (Fig. 3*C*). Conversely, brief DAG increases caused by repeated 5-s applications of 0.1 μm P2Y_1_ receptor agonist MRS 2365 caused transient translocations of PKCϵ to and from the plasma membrane, whereas continuous application of the drug induced sustained responses (Fig. 3*D*).

**FIGURE 4.**
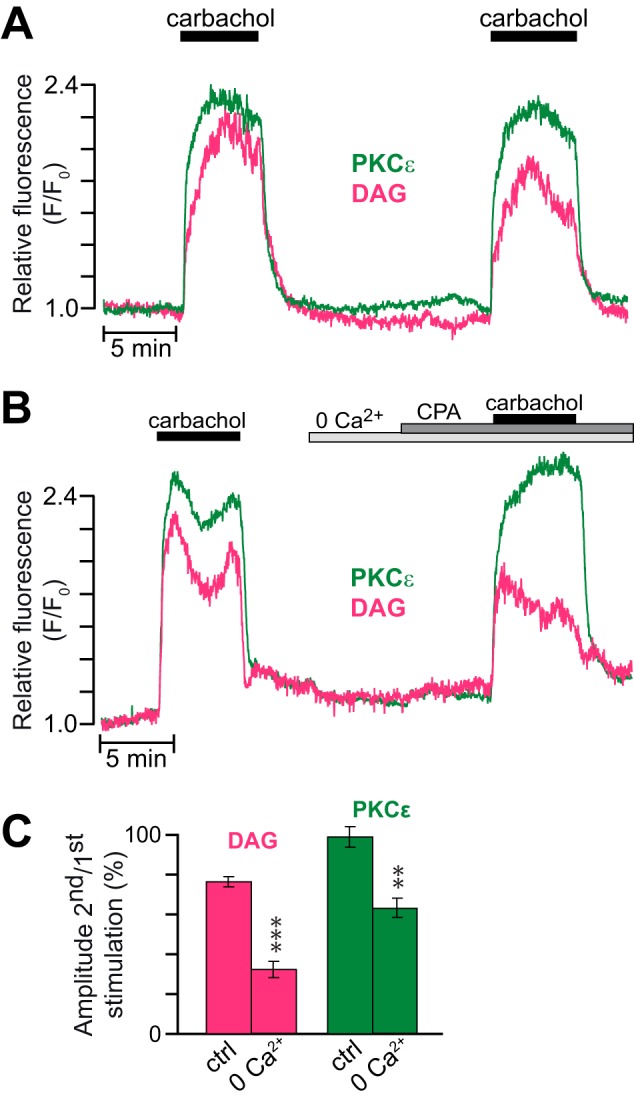
**The carbachol-induced translocation of PKCϵ reflects DAG dynamics.** *A*, DAG dynamics and PKCϵ translocation in response to repeated stimulations with 100 μm carbachol in the presence of 3 mm glucose. *B*, a similar recording but with the second stimulation made after omission of extracellular Ca^2+^ and addition of 2 mm EGTA and 50 μm cyclopiazonic acid (*CPA*). *C*, means ± S.E. for the relative amplitudes of DAG and PKCϵ translocation during two consecutive stimulations with carbachol in the presence or absence of Ca^2+^ as in *A* and *B* (*n* = 25 cells from three experiments and 14 cells from two experiments, respectively). **, *p* < 0.0007; ***, *p* < 3 × 10^−5^ for the difference from the control (*ctrl*).

The stable acetylcholine analogue carbachol activates phospholipase C, and the resulting increases in DAG and cytoplasmic Ca^2+^ concentrations induce PKC activation. Two 5-min periods of carbachol stimulation 15 min apart resulted in comparable plasma membrane DAG increases and PKC translocation dynamics (Fig. 4*A*). PKCϵ-GFP translocation to the plasma membrane was sustained and typically very similar to the DAG dynamics (Fig. 4*A*). Omission of Ca^2+^ from the extracellular medium together with addition of EGTA and the Ca^2+^-ATPase inhibitor cyclopiazonic acid to prevent elevations of the cytoplasmic Ca^2+^ concentration reduced carbachol-induced DAG production, probably as a result of elimination of positive feedback from Ca^2+^ on phospholipase C (31), and PKCϵ-GFP translocation was consequently also slightly reduced (Fig. 4, *B* and *C*). These findings show that the glucose-induced DAG microdomains are associated with translocation of nPKCs to the plasma membrane and reinforce the notion that DAG is sufficient for driving translocation of nPKCs.

##### Glucose Induces Complex Oscillatory cPKC Translocation Reflecting both Ca^2+^ and DAG Dynamics

**FIGURE 5.**
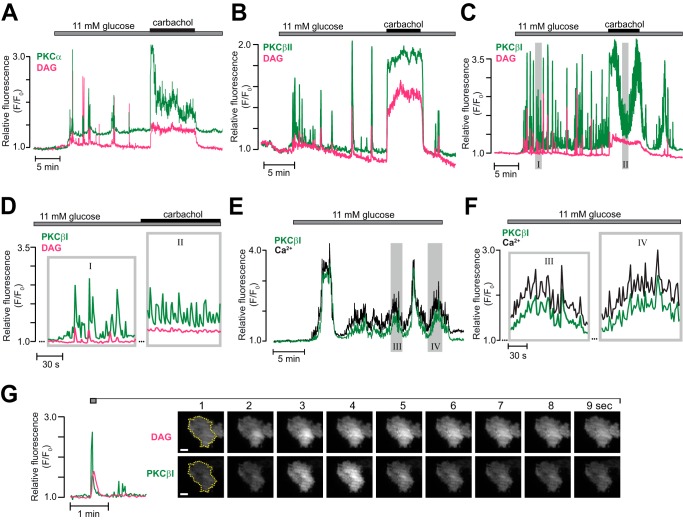
**Glucose- and carbachol-induced translocation of cPKC isoforms.** *A–C*, simultaneous TIRF recordings of DAG (*magenta*) and GFP-tagged PKC (*green*) of the α (*A*, *n* = 8 cells from three experiments), βII (*B*, *n* = 6 cells from two experiments), or βI (*C*, *n* = 29 cells from five experiments) isoforms in MIN6 cells stimulated by an increase in glucose concentration from 3 to 11 mm followed by addition of 100 μm carbachol. The regions highlighted by *shaded rectangles* are shown on an expanded time basis in *D. D*, time expansions of the recording from *C. E*, slow and fast oscillations of [Ca^2+^]_pm_ and concomitant PKCβI translocation during a step increase in glucose concentration from 3 to 11 mm (representative of seven of 14 cells in six experiments). The regions highlighted by *shaded rectangles* are shown on an expanded time basis in *F. F*, time expansions of the recording from *E* showing fast oscillations of PKCβI translocation and [Ca^2+^]_pm_ superimposed on slower ones. *G*, TIRF intensity recording and corresponding image pairs from the period indicated in the graph. The images were acquired every second and show that the DAG spike occurs in a restricted part of the plasma membrane and that PKCβI translocates to the same region. *Scale bars* = 5 μm.

Experiments were also performed with MIN6 cells co-expressing the DAG biosensor and either of the GFP-tagged cPKC isoforms PKCα, βI, or βII. Elevation of the glucose concentration to 11 mm induced prominent, repetitive translocation of all three cPKC isoforms (Fig. *5*, *A–C*). The translocation pattern of PKCβII differed slightly from that of PKCα and PKCβI. Of 10 cells expressing PKCβII-GFP, four showed DAG increases and PKC translocation even at a substimulatory glucose concentration (data not shown). In the remaining cells, 11 mm glucose induced prominent, repetitive translocation of PKCβII to the plasma membrane with kinetics strikingly similar to that of DAG, and few glucose-induced PKCβII recruitments to the membrane occurred without a concomitant DAG spike (Fig. 5*B*). Carbachol triggered pronounced and sustained recruitment of PKCβII to the plasma membrane, mirroring the DAG dynamics (Fig. 5*B*).

The translocation pattern of PKCβI consisted of a small, sustained increase of fluorescence with superimposed, very pronounced (>3-fold increases in fluorescence) repetitive translocation peaks that only partially reflected parallel DAG spiking (Fig. 5, *C* and *D*). Although carbachol transformed the DAG pattern to a sustained increase, PKCβI continued to show fast, repetitive translocation from an elevated level that sometimes fluctuated slowly (Fig. 5, *C* and *D*). The fast PKCβI translocations that did not coincide with DAG spikes are most likely driven by rapid glucose-induced changes in the cytoplasmic Ca^2+^ concentration. Fig. 5, *E* and *F*, shows an example of a glucose-stimulated cell with slow oscillations of PKCβI translocation (duration >1 min) and superimposed spiking almost perfectly mirroring slow and fast oscillations of [Ca^2+^]_pm_. With PKCβI, it was more difficult to observe spatially restricted translocation coinciding with DAG microdomains than it was with the nPKCs. This was because PKCβI translocation spikes were so frequent and clearly dependent on factors in addition to DAG. Fig. 5*G* shows one of the rather infrequent examples of an isolated PKCβI translocation event paralleled by local DAG generation.

**FIGURE 6.**
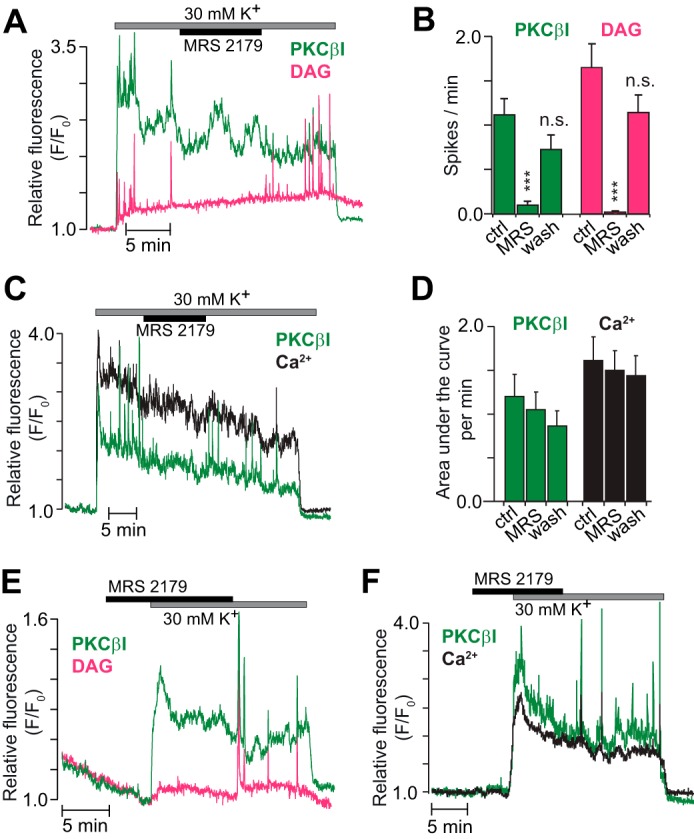
**The translocation pattern of cPKCs reflects both DAG and Ca^2+^.** *A*, DAG dynamics (*magenta*) and PKCβI translocation (*green*) in a single MIN6 cell exposed to 3 mm glucose, 30 mm K^+^, and 10 μm of the P2Y_1_ receptor antagonist MRS 2179. *B*, means ± S.E. for the frequency of PKCβI and DAG spikes during K^+^ depolarization in the presence of MRS 2179 and after washout of the drug (*n* = 9 cells from four experiments). ***, *p* < 0.001 for the difference from the high K^+^ control. *ns*, not significant. *C*, PKCβI translocation (*green*) and [Ca^2+^]_pm_ (*black*) in a single cell stimulated as in *A. D*, means ± S.E. for the area under the curve per minute of PKCβI translocation and [Ca^2+^]_pm_ before, during, and after addition of MRS 2179 from experiments as in *C* (*n* = 12 cells from three experiments). *E* and *F*, similar experiments as in *A* and *C* but with MRS 2179 present before exposure to 30 mm K^+^. *n* = 10 cells from three experiments (*E*) and 15 cells from three experiments (*F*).

Membrane depolarization with a high K^+^ concentration resulted in sustained plasma membrane translocation of PKCβI-GFP with superimposed spiking (Fig. 6*A*). This PKCβI-GFP spiking was inhibited when DAG spiking was prevented by P2Y_1_ receptor inhibition with MRS 2179 (Fig. 6, *A* and *B*). However, the sustained translocation of PKCβI-GFP was unaffected by the drug (Fig. 6*A*), showing dynamics strikingly similar to those of [Ca^2+^]_pm_, with a slight time-dependent decline (Fig. 6, *C* and *D*). Likewise, introduction of MRS 2179 before K^+^ depolarization did not affect the subsequent [Ca^2+^]_pm_ elevation and stable PKCβI translocation but prevented the appearance of rapid DAG and [Ca^2+^]_pm_ spikes as well as the concomitant transient PKCβI translocations (Fig. 6, *E* and *F*).

**FIGURE 7.**
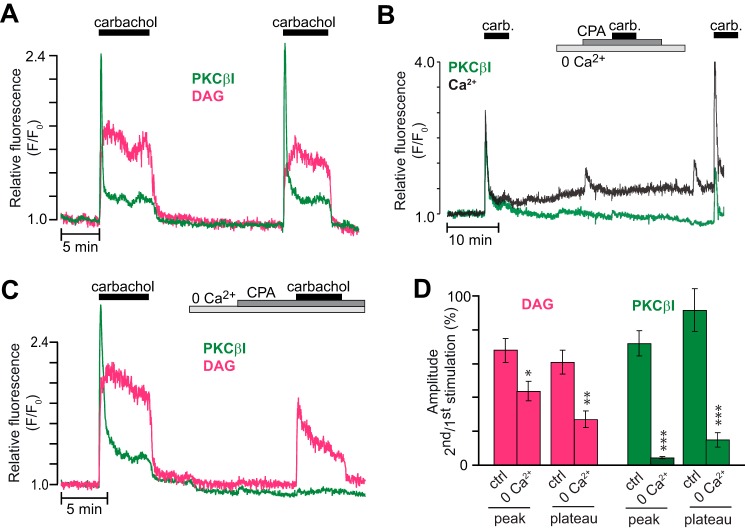
**Ca^2+^ is required for muscarinic receptor-induced translocation of cPKCs.** *A*, DAG dynamics (*magenta*) and PKCβI translocation (*green*) in response to repeated stimulations with 100 μm carbachol in the presence of 3 mm glucose. *B*, parallel recordings of [Ca^2+^]_pm_ (*black*) and PKCβI translocation (*green*) during exposure to carbachol (*carb.*) under control and Ca^2+^-deficient conditions (*n* = 22 cells from five experiments). *CPA*, cyclopiazonic acid. *C*, similar as in *A*, but extracellular Ca^2+^ was omitted, and 2 mm EGTA and 50 μm cyclopiazonic acid were added before the second carbachol stimulation. *D*, means ± S.E. for the relative amplitudes of the peak and following plateau of simultaneously recorded DAG production and PKCβI translocation in response to two consecutive carbachol applications in the presence or absence of Ca^2+^ (*n* = 18 and 10 cells from three and two experiments for control and 0 Ca^2+^, respectively). *, *p* < 0.05; **, *p* < 0.0007; ***, *p* < 3 × 10^−5^ for the difference from the control (*ctrl*).

In the presence of 3 mm glucose, carbachol triggered an initial distinct peak of PKCβI-GFP translocation to the plasma membrane, followed within a few seconds by dissociation and stabilization at a sustained plateau corresponding to 15% ± 3% of the peak translocation (*n* = 19; Fig. 7, *A* and *B*). Similar kinetics were not always observed for DAG production, and the plateau phase corresponded to 83% ± 5% of the peak amplitude (*n* = 19, Fig. 7*A*). PKCβI translocation dynamics were reminiscent of those of [Ca^2+^]_pm_, which were dominated by a pronounced initial peak caused by inositol-1,4,5-trisphosphate-mediated mobilization of Ca^2+^ from the endoplasmic reticulum, followed by a modest stable elevation, mainly reflecting store-operated Ca^2+^ entry (Fig. 7*B*) (32). The sustained PKCβI-GFP translocation probably reflects the Ca^2+^ dependence of DAG binding (33, 34). Omission of Ca^2+^ from the extracellular medium together with addition of EGTA and cyclopiazonic acid prevented the carbachol-induced rise of [Ca^2+^]_pm_ (Fig. 7*B*) and effectively prevented plasma membrane translocation of PKCβI-GFP (Fig. 7, *C* and *D*). Our findings reinforce the notion that the translocation of cPKCs is strictly Ca^2+^-dependent but also emphasize that DAG mediates a fast and transient component of membrane translocation.

##### The Atypical Isoform PKCζ Does Not Translocate to the Plasma Membrane in Response to Muscarinic Receptor Activation, Glucose, or Insulin

**FIGURE 8.**
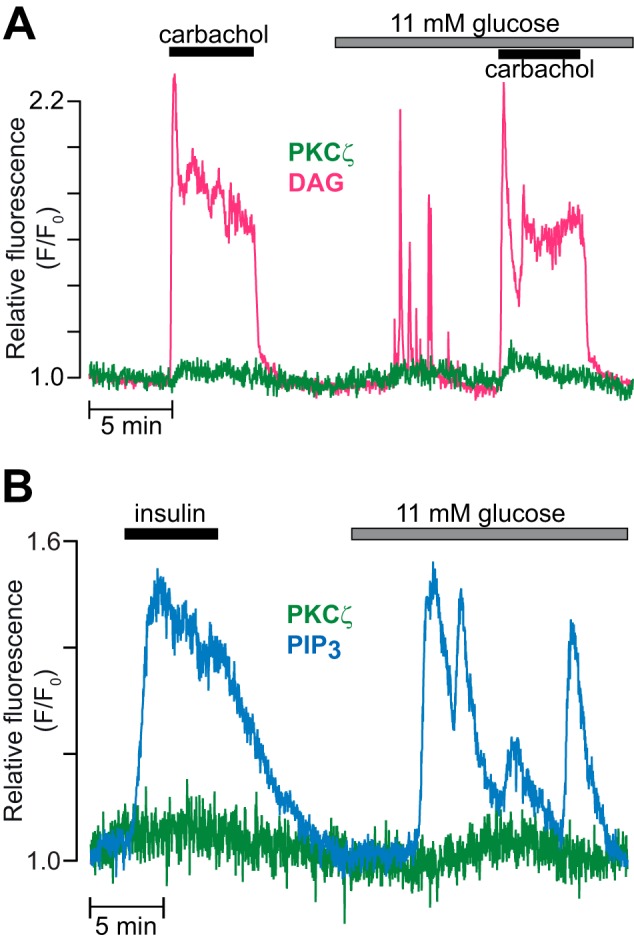
**The atypical isoform PKCζ does not translocate to the plasma membrane in response to muscarinic receptor activation, glucose, or insulin.** *A*, TIRF recording of DAG (*magenta*) and PKCζ-GFP (*green*) in a single MIN6 cell stimulated with 100 μm carbachol in the presence of 3 and 11 mm glucose (*n* = 11 cells from three experiments). *B*, TIRF recording of phosphatidylinositol 3,4,5-trisphosphate (*PIP_3_*, *blue*) and PKCζ-GFP (*green*) in a MIN6 cell during exposure to 300 nm insulin and an increase in glucose concentration from 3 to 11 mm (*n* = 7 cells for 100 nm and 13 for 300 nm insulin in two experiments).

We also investigated the translocation dynamics of PKCζ, an aPKC family member expressed in β cells (5–10). Although 100 μm carbachol or rise of glucose elicited pronounced DAG signaling, there was no consistent PKCζ-GFP translocation to the plasma membrane (Fig. 8*A*). A weak tendency of carbachol to increase PKCζ-GFP membrane fluorescence was probably unspecific and reproduced in control experiments with plasma membrane-anchored GFP (data not shown). Because PKCζ-GFP might be responsive to phosphatidylinositol 3,4,5-trisphosphate, we investigated the effect of insulin. At 100–300 nm, insulin induced a phosphatidylinositol 3,4,5-trisphosphate increase of similar magnitude as glucose but without recruitment of PKCζ-GFP to the plasma membrane (Fig. 8*B*).

##### Inhibition of P2Y_1_ Receptors Suppresses Glucose-induced Insulin Secretion

**FIGURE 9.**
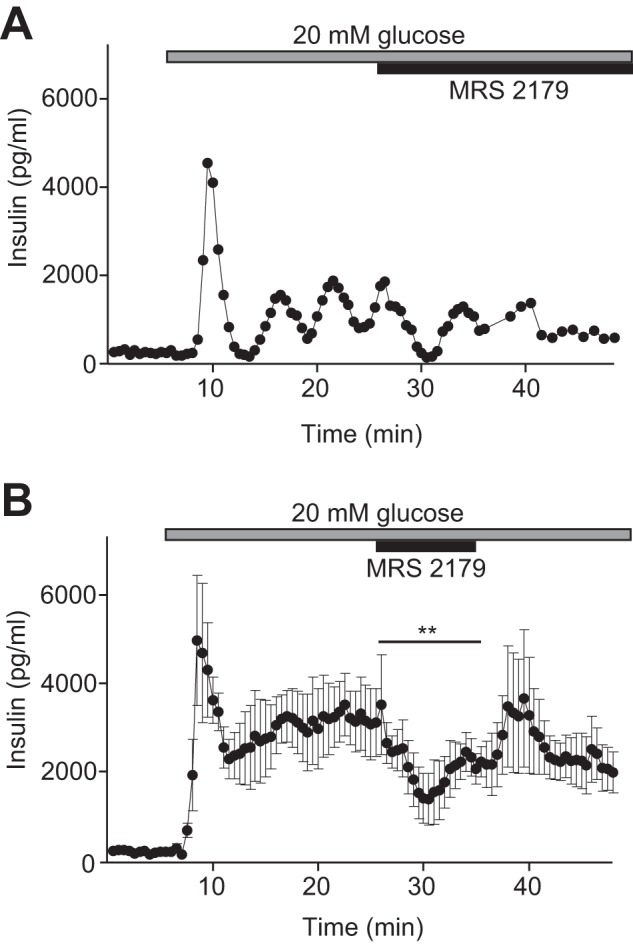
**P2Y_1_ receptor inhibition suppresses glucose-induced pulsatile insulin secretion.** *A*, example recording of insulin secretion from a group of 20–24 superfused MIN6 pseudoislets during elevation of the glucose concentration from 3 to 20 mm and subsequent exposure to 10 μm MRS 2179. Samples were collected every 30 s for the first 38.5 min and then every 60 s. *B*, means ± S.E. from five recordings of insulin secretion in 30-s fractions from MIN6 pseudoislets exposed to 3 or 20 mm glucose and 10 μm MRS 2179. **, *p* < 0.01 refers to the difference in time-average secretion during exposure to MRS 2179 compared with that for the preceding 10-min control period.

To clarify whether DAG spiking and PKC translocation contributed to glucose-induced insulin release, we measured insulin secretion dynamics from MIN6 pseudoislets. Increase of glucose from 3 to 20 mm induced pulsatile insulin release that deteriorated after introduction of 10 μm MRS 2179 (Fig. 9*A*). After averaging data from five experiments, pulsatility was no longer evident, but there was significant suppression of insulin release during MRS 2179 exposure (time-average secretion, 64% ± 9% of the preceding 10-min control period; *p* < 0.01; Fig. 9*B*).

## Discussion

This study demonstrates that exocytosis-induced microdomains of DAG recruit both conventional and nPKCs to the plasma membrane in β cells. Autocrine signaling is thus involved in controlling the spatiotemporal dynamics of PKC activity in glucose-stimulated β cells. In contrast to the view that DAG changes slowly and that Ca^2+^ accounts for the rapid translocation of PKC (28, 35), this study highlights that fast, oscillatory PKC translocation can be determined by DAG spiking, whereas more sustained translocation is Ca^2+^-dependent.

PKC translocation dynamics has previously been investigated in insulin-secreting β cells. However, glucose-induced plasma membrane recruitment of PKCβI-GFP has not been observed before, probably because of insufficient time resolution and difficulties to specifically detect changes in plasma membrane fluorescence (25). PKCα and PKCβII have been reported to undergo oscillatory membrane translocation in glucose-stimulated primary β cells (18, 23), probably reflecting slow oscillations of the free cytosolic Ca^2+^ concentration (23, 36). Endogenous PKCα has been found to strongly associate with the plasma membrane during the first phase of insulin secretion, followed by a dissociation and subsequent reassociation during second-phase insulin release (10). We now discovered more complex and rapid translocation dynamics that were strikingly parallel with DAG spiking. We propose that the previously described translocation dynamics merely reflect the Ca^2+^-driven component of cPKC activation and that improved temporal resolution and selective imaging of the plasma membrane now reveal a superimposed component of transient, rapid, and local DAG-mediated plasma membrane binding of cPKCs. Subtle variations in translocation patterns among closely related isoforms indicate that the PKCs are fine-tuned to respond differently to distinctive DAG and Ca^2+^ signals.

Although DAG-dependent activation of nPKCs is well established, it is notable that all tested isoforms were capable to respond to glucose-induced DAG spiking with strikingly rapid translocation to the plasma membrane. Several previous studies failed to detect glucose-induced plasma membrane association of PKCδ (10, 36, 37), and in HEK293 cells, this isoform translocates to the endoplasmic reticulum after activation of purinergic receptors (38). Another study stressed a difference between the δ and ϵ isoforms, with PKCϵ translocating to the cell periphery and PKCδ to perinuclear sites in response to glucose (21). The presently observed similarity in plasma membrane translocation dynamics of the two isoforms was therefore somewhat surprising, especially because PKCδ and ϵ often have different or even opposing, effects (39). However, a similar spatiotemporal pattern at the plasma membrane does not exclude distinct activation at other subcellular sites. Endogenous PKCϵ was reported to associate with insulin staining near the nucleus, and glucose was found to induce changes in staining intensity, reflecting the biphasic insulin response of the perfused rat pancreas (10). Although the spatial resolution in the latter study did not permit a definite localization of the staining to the secretory granules, confocal imaging of PKCϵ-GFP supported a glucose-induced association of PKCϵ to insulin granules in INS1E insulinoma cells, with the most prominent effect near the plasma membrane (25). No granular pattern was evident in the presently observed PKCϵ distribution, indicating that the protein also can interact directly with the plasma membrane.

PKCη differed from the other nPKCs in that transient membrane translocation sometimes occurred without a simultaneously detected DAG elevation. The reason may be a higher DAG affinity of the C1 domain of PKCη compared with that of the PKCγ-derived DAG sensor (27). PKCη has been found previously to locate to the cytoplasm in rat islets (5) and to membranes in RINm5F cells (8), but, unlike in this study, carbachol did not affect the distribution pattern in either case.

The aPKCs lack a Ca^2+^-binding C2 domain, and their C1 domain variant is unable to bind DAG (1, 2). The carbachol-induced membrane translocation is therefore likely mediated by protein-protein interactions via the PB1 domain, which is specific for aPKCs (1, 2). Carbachol-induced translocation of PKCζ to the plasma membrane has been detected previously by Western blotting and suggested to mediate carbachol-stimulated insulin release in RINm5F cells (8). Another study using various PKC inhibitors concluded that glucose-induced insulin secretion is, at least in part, dependent on activation of an aPKC isoform (20). Using immunohistochemistry, Warwar *et al.* (10) demonstrated that glucose induces transient translocation of PKCζ to the plasma membrane, corresponding to first-phase insulin secretion, and that prolonged stimulation led to accumulation of PKCζ in the nucleus. The present findings do not support the view that glucose or carbachol cause rapid association of PKCζ with the plasma membrane but do not allow conclusions about its localization or activity in other subcellular compartments.

Our findings strengthen the idea that DAG spiking underlies the secretagogue-induced, repetitive, brief plasma membrane associations of novel and cPKCs. They also emphasize the requirement of DAG production for nPKC translocation and suggest that cPKCs can associate with the plasma membrane without prominent increases in DAG concentration. The modest, stable DAG elevation caused by membrane depolarization in the presence of MRS 2179 (Figs. 3*B* and 6*D*) probably results from Ca^2+^-driven phospholipase C activity (29, 40–42). Membrane binding of cPKCs is also mediated via interaction of the Ca^2+^-binding C2 domain with other lipids, primarily phosphatidylserine, phosphatidylinositol-4,5-bisphosphate, and phosphatidylinositol 3,4,5-trisphosphate (43, 44). The C2 domain of nPKCs is not required for membrane binding. Instead, their C1 domain has an increased affinity for DAG (33). Moreover, the C1 domain binds selectively and stereospecifically to phosphatidylserine-containing membranes in a DAG-dependent manner (33, 34), which is consistent with our finding that PKCϵ did not translocate when DAG spiking was prevented.

The very short periods of PKC activation may explain why it has been difficult to detect increased PKC-mediated phosphorylation in glucose-stimulated β cells (18). Our data clearly show that a brief DAG spike with concomitant PKC translocation is sufficient for inducing protein phosphorylation. In β cells, the MARCKS protein seems to be phosphorylated primarily by an nPKC. The transient nature of these PKC signaling events should be suitable for regulation of rapid processes such as exocytosis. Indeed, several components of the exocytosis machinery, including SNAP25 and Munc18, are targets for PKC (45, 46). Studies of the effects of PKC inhibitors on insulin secretion have nevertheless yielded contradictory results (18–23). The inconsistencies may in part be explained by different effects of PKC on the initial and late phases of secretion (23). Another possibility is that PKC is important for the periodic pattern of insulin release. Accordingly, inhibition of the purinergic feedback mechanism that underlies the DAG spiking and transient PKC translocation markedly perturbed pulsatile insulin secretion (Fig. 9). Similar findings have been reported previously from the perifused rat pancreas using a conventional immunoassay (47) and from isolated mouse insulinoma cells using a single-cell optical assay (29). The disrupted pulsatility was associated with either inhibition (Ref. 29 and this study) or stimulation (47) of insulin secretion, a discrepancy that is probably due to differences in experimental preparations. The effects of adenine nucleotides on β cells are pleiotropic and involve the action of several purinergic receptors (reviewed in Refs. 48, 49). Recent work in human β cells confirmed that P2Y_1_ receptors mediate autocrine stimulation of insulin secretion (50). Further investigations are required to define the functional importance of the various PKC isoforms in β cells. In conclusion, insulin secretagogues induce transient DAG microdomains that rapidly recruit both conventional and novel PKCs to the β cell plasma membrane. The findings implicate PKC signaling in the autocrine regulation of β cell function and emphasize a role of DAG for rapid kinetic control of PKC-dependent cellular processes.

## Experimental Procedures

### 

#### 

##### Reagents and DNA Constructs

MRS 2179 tetrasodium salt, MRS 2365, Gö 6976, and Gö 6983 were purchased from Tocris Bioscience (Bristol, UK). Bovine serum albumin was from Roche Diagnostics and physiological salts for the experimental buffer from Merck. Cyclopiazonic acid, EGTA, and HEPES were purchased from Sigma-Aldrich (St. Louis, MO). The plasmids containing GFP-labeled PKCs and MARCKS-GFP were provided by Prof. Tobias Meyer (Stanford University) and Prof. Naoaki Saito (Kobe University), respectively. A DAG biosensor based on the C1 domain tandem repeat in PKCγ (γC1aC1b-mCherry) was created as described earlier (29), and the genetically encoded Ca^2+^ sensor R-GECO (51) was used for measurements of the cytoplasmic Ca^2+^ concentration. mCherry targeted to the plasma membrane by a CAA*X* motif was used as plasma membrane marker.

##### Cell Culture and Transfection

If not otherwise stated, all cell culture reagents were from Life Technologies. Insulin-secreting MIN6 insulinoma cells (30) of passages 17–31 were cultured in DMEM containing 25 mm glucose and supplemented with 2 mm glutamine, 70 μm 2-mercaptoethanol, 100 units/ml penicillin, 100 μg/ml streptomycin, and 15% fetal calf serum and kept at 37 °C in a humidified atmosphere with 5% CO_2_. Cells were transfected while being seeded onto 25-mm coverslips (Menzel-Gläser, Thermo Fisher Scientific, Waltham, MA) coated with polylysine (0.01 mg/ml). For each coverslip, ∼0.2 million cells were suspended in 100 μl of Opti-MEM® medium containing 0.5 μl of Lipofectamine^TM^ 2000 with up to 0.3 μg of plasmid DNA and plated onto the glass. After 3 h, when the cells were attached, the transfection was interrupted by addition of 3 ml of complete culture medium. Experiments were conducted after 13–36 h of further culture. For insulin secretion experiments, 1.5 million MIN6 cells were allowed to form pseudoislets by culture in a 60-mm polystyrol Petri dish (Sarstedt, Nümbrecht, Germany) for 4 days.

##### TIRF Microscopy Recordings of [Ca^2+^]_pm_, DAG, and PKC Translocation

Before each experiment, the coverslip with attached cells was transferred to experimental buffer and incubated for 30 min at 37 °C. The buffer contained 125 mm NaCl, 4.8 mm KCl, 1.3 mm CaCl_2_, 1.2 mm MgCl_2_, 25 mm HEPES, 3 mm d-glucose, and 0.1% (w/v) bovine serum albumin with the pH adjusted to 7.4 with NaOH. After preincubation, the coverslip with the cells was mounted in an open 50-μl chamber and superfused with buffer at a rate of 0.3 ml/min. All experiments were performed at 37 °C.

The plasma membrane localization of GFP-tagged PKCs and the DAG translocation biosensor as well as the intensity of the Ca^2+^ sensor fluorescence were measured with a TIRF microscopy setup consisting of an Eclipse Ti microscope (Nikon) with a TIRF illuminator and a ×60, 1.45 numerical aperture objective. The 488-nm line of a multiline argon laser (ALC 60X, Creative Laser Production, Munich, Germany) and the 561-nm line from a diode-pumped solid-state laser (Jive, Cobolt AB, Solna, Sweden) were selected by narrow bandpass filters (Semrock, Rochester, NY) in a filter wheel (Sutter Instruments, Novato, CA) and used for excitation of GFP (488 nm), mCherry, and R-GECO (561 nm). Fluorescence was detected with a back-illuminated DU-897 electron multiplying charge-coupled device camera (Andor Technology, Belfast, Northern Ireland) controlled by MetaFluor software (Molecular Devices Corp., Downington, PA). Emission wavelengths were selected with a filter wheel (Sutter Instruments) and the following filters: 527/27-nm half-bandwidth for GFP and 584-nm long pass for mCherry and R-GECO (Semrock Rochester, NY). For time-lapse recordings, images or image pairs were acquired every 1 s. The laser beam was blocked by a shutter (Sutter Instruments) between image captures to minimize exposure to the potentially harmful light.

##### Measurements of Insulin Secretion

Groups of 20–24 pseudoislets were placed in a 20-μl chamber superfused at 130 μl/min with a similar experimental buffer as described above but with 2.6 mm CaCl_2_. After 35 min of superfusion with buffer containing 3 mm glucose, the perifusate was collected in 30- or 60-s fractions during elevation of the glucose concentration to 20 mm and addition of MRS 2179. The collected medium was immediately put on ice, and after appropriate dilution, insulin was measured using a human insulin AlphaLISA immunodetection kit (PerkinElmer Life Sciences) according to the protocol of the manufacturer.

##### Data Analysis

Fluorescence intensities were logged from individual cells as a function of time using MetaFluor (Molecular Devices) and expressed relative to the initial fluorescence after subtraction of background (F/F_0_). The response amplitudes and area under the curve were evaluated using Igor Pro software (Wavemetrics Inc.) and are presented as mean ± S.E. Statistical analysis was performed with paired or two-sample equal variance Student's *t* test as appropriate.

## Author Contributions

A. W. and A. T. designed the study and wrote the paper. A. W. and Q. Y. performed the experiments. All authors analyzed the data and approved the final version of the manuscript.

## Footnotes

2 The abbreviations used are:

cPKC: conventional PKC
DAG: diacylglycerol
nPKC: novel PKC
aPKC: atypical PKC
MARCKS: myristoylated alanine-rich kinase C substrate
[Ca^2+^]_pm_: cytoplasmic Ca^2+^ concentration beneath the plasma membrane
TIRF: total internal reflection fluorescence.
